# Long-term Maintenance of CD4 T Cell Memory Responses to Malaria Antigens in Malian Children Coinfected with *Schistosoma haematobium*

**DOI:** 10.3389/fimmu.2017.01995

**Published:** 2018-02-01

**Authors:** Kirsten E. Lyke, Abdoulaye Dabo, Charles Arama, Issa Diarra, Christopher V. Plowe, Ogobara K. Doumbo, Marcelo B. Sztein

**Affiliations:** ^1^Center for Vaccine Development, Institute for Global Health, School of Medicine, University of Maryland, Baltimore, MD, United States; ^2^Division of Malaria Research, Institute for Global Health, School of Medicine, University of Maryland, Baltimore, MD, United States; ^3^Malaria Research and Training Center, International Centers for Excellence in Research (NIH), University of Science Techniques and Technologies of Bamako, Bamako, Mali

**Keywords:** malaria, schistosomiasis, coinfection, multifunctional T cells, T cell memory, memory, *Plasmodium falciparum*

## Abstract

Polyparasitism is common in the developing world. We have previously demonstrated that schistosomiasis-positive (SP) Malian children, aged 4–8 years, are protected from malaria compared to matched schistosomiasis-negative (SN) children. The effect of concomitant schistosomiasis upon acquisition of T cell memory is unknown. We examined antigen-specific T cell frequencies in 48 Malian children aged 4–14 to a pool of malaria blood stage antigens, and a pool of schistosomal antigens, at a time point during a malaria episode and at a convalescent time point ~6 months later, following cessation of malaria transmission. CD4^+^ T cell-derived memory responses, defined as one or more significant cytokine (IFN-γ, TNF-α, IL-2, and/or IL-17_A_) responses, was measured to schistoma antigens in 18/23 SP children at one or both time points, compared to 4/23 SN children (*P* < 0.0001). At the time of malaria infection, 12/24 SN children and 15/23 SP children (*P* = 0.29) stimulated with malaria antigens demonstrated memory recall as defined by CD4-derived cytokine production. This compares to 7/23 SN children and 16/23 SP children (*P* = 0.009) at the convalescent timepoint. 46.2% of cytokine-producing CD4^+^ T cells expressed a single cytokine after stimulation with malaria antigen during the malaria episode. This fell to 40.9% at follow-up with a compensatory rise of multifunctional cytokine secretion over time, a phenomenon consistent with memory maturation. The majority (53.2–59.5%) of responses derived from CD45RA^−^CD62L^−^ effector memory T cells with little variation in the phenotype depending upon the time point or the study cohort. We conclude that detectable T cell memory responses can be measured against both malaria and schistosoma antigens and that the presence of *Schistosoma haematobium* may be associated with long-term maintenance of T memory to malaria.

## Introduction

*Plasmodium falciparum* and *Schistosoma* spp. are co-endemic parasitic diseases with worldwide distribution. Revised estimates suggest that falciparum malaria causes 212 million infections annually, while schistosomiasis affects an estimated 207 million with 92% residing in Africa (1, 2). Both parasitic diseases predominate in sub-Saharan Africa but the host impact of dual infection is understudied. A growing body of evidence suggests that a pre-existing infection can modulate the effects of a second infection within the human host. This can occur indirectly, as is the case in HIV where a reduction in CD4^+^ T cells results in host susceptibility to viral and parasitic infections. This can also occur directly, such as in helminth infections, where chronicity of infection and the robust response of the immune system results in a background environment that modulates the host response to a secondary infection. The term, “helminth” encompasses a wide range of representative examples including soil transmitted helminthes with limited systemic perturbation, as well as tissue-invasive helminths capable of surviving for years within the human host. *Schistosoma haematobium* is a long-lived blood fluke capable of exerting a persistent stimulatory effect on the host immune system, chiefly to egg antigens, and modestly protects against clinical uncomplicated *P. falciparum* malaria in an age-specific manner in West African children (3, 4).

Chronic schistosoma infection, characterized by persistent egg patency, results in an expansion of T_H_2-mediated responses (5, 6), as well as the induction of regulatory pathways leading to parasite immunomodulation (7). While the immunologic mechanisms involved in exerting clinical protection may be multifactorial, there is evidence of differential immunologic responses to malaria in children with underlying *S. haematobium* (8–13). These immunologic perturbations may result in an altered clinical response to an acute malaria exposure. A systematic examination of the immune response to malaria among Malian children with asymptomatic *S. haematobium* demonstrate significantly more memory B cell response to pooled malaria vaccine antigens [apical membrane antigen 1 (AMA1) and merozoite surface protein 1 (MSP1)] (14), altered cytokine patterns characterized by IL-4 and IL-10 T_H_2-enrichment as well as IL-6 and IFN-γ elevation (8), and suppressed T regulatory cells response (15), compared to age-matched children without underlying schistosomiasis.

Protection against pre-erythrocytic malaria may be mediated by CD4 T cells as evidenced by the ability for circumsporozoite-specific CD4 T cells to mediate clearance of hepatocyte infection in murine models and adoptive transfer of protection *in vivo* (16, 17). Multifunctionality, a term referring to the ability of T cells to simultaneously produce multiple cytokines (e.g., IFN-γ, IL-2, and TNF-α) has been associated with increased protective responses against some infections and is therefore generally accepted as an indicator of the “quality” of T cell response (18, 19). CD4 T cells may gain effector function with maturation resulting in the ability to secrete multiple cytokines while eventually losing effector function in a linear manner (19). Evidence of durable immunologic memory to malaria antigens is mixed, particularly in young children, where repetitive and ongoing exposure to malaria is required to achieve and maintain immunity (20, 21). Chronic immune activation due to a helminth infection (characterized by a T_H_2 cytokine production pattern) may alter responses to a secondary stimuli that depend upon T_H_1 cytokine production, such as malaria antigens (5, 22), as well as affect the induction and maintenance of T cell memory responses (23). For example, it has been reported that underlying filarial infection suppresses malaria-specific T_H_1 and T_H_17 responses in co-infected Malian children (24). We report herein, findings that demonstrate maintenance of CD4 T cell memory responses at a convalescent time point following documentation of acute malaria infection in children with underlying *S. haematobium* compared to helminth-free, age-matched children.

## Materials and Methods

### Study Population and Clinical Trial Design

Bandiagara (pop. ~13,600) is located in Mali, West Africa, and has intense seasonal transmission (July–December) of *P. falciparum* malaria. The entomologic inoculation rate at the time of this field study ranged from 20 to 60 infected bites per month at peak transmission and, children experience a mean of 1.54 symptomatic malaria episodes per season (25). *S. haematobium* and *Schistosoma mansoni* are endemic to the area (26, 27), with *S. haematobium* prevalence of 25% in children aged 4–14 years and ~50% in adults (3). Children can be exposed to schistosomes as early as age 2 or 3 years. This study was conducted over two sequential malaria transmission seasons (2002–03) and study details have previously been reported (3, 8). Briefly, children aged 4–14 years of age, diagnosed as having asymptomatic, chronic *S. haematobium* (SP), were age, gender, and residence-matched to a child without schistosomiasis [schistosomiasis-negative (SN)] prior to malaria transmission. Children were followed weekly over the malaria transmission season (25 weeks) and at a dry season follow-up appointment (~6–9 months after enrollment at a time when standing water pools had dried and schistosoma transmission had ceased). The primary endpoint of the clinical trial was time to first clinical malaria infection. Clinic personnel were available 24 h-a-day throughout study duration to detect, examine, and treat symptomatic malaria episodes. A clinical episode of malaria was defined as *P. falciparum* parasitemia and axillary temperature ≥37.5°C on active surveillance, or parasitemia and symptoms leading to treatment-seeking behavior in the absence of other clear cause on passive surveillance. All children were pre-treated with albendazole to eliminate concomitant helminth infections and study samples were drawn at the time of their first clinical malaria episode (or at study week 25/Day 175 in the absence of a clinical infection) and again at the final dry season appointment. Children were optimally treated for schistosomiasis with praziquantel at the final appointment.

### Ethics

This trial was carried out in accordance with the recommendations of the University of Bamako and the University of Maryland Institutional Review Boards (IRBs) with written consent obtained from all legal guardians. Village permission to conduct research was obtained from village chiefs, government officials and traditional healers prior to study initiation. Individual written informed consent was obtained from the parent or legal guardian of each child prior to screening and enrollment in accordance with the Declaration of Helsinki. All children displaying gross hematuria or symptoms of genitourinary pathology were treated with praziquantel (40 mg/kg) therapy and discharged from the study.

### Sample Collection

Patient whole blood (7–10 mL) was collected at the study clinic into sterile eppendorf and EDTA tubes on admission, prior to institution of anti-malarial therapy, and immediately refrigerated. Sera was processed as previously described (8). Blood was processed by density centrifugation, within 2 h of acquisition, utilizing lymphocyte separation medium (ICN Biomedical Inc, Aurora, OH, USA) following standard techniques (28). Peripheral blood mononuclear cells (PBMC) were resuspended in media and linear-rate frozen using isopropyl alcohol containers (Nalgene, USA) to −70°C in the field site before transfer in liquid nitrogen storage containers to the University of Maryland at Baltimore. Samples for the primary experiment were chosen based on the availability of both a transmission and dry season time point *and the presence of at least 10* × *10^6^ cells* for each child at each time point. A secondary analysis from the same study, which was part of a larger, previously unpublished set of experiments, examined samples with only a single transmission season time point utilizing a second flow cytometry panel of antibodies.

### PBMC Stimulation

Each primary experiment consisted of a U.S. malaria-naïve adult control, and paired samples (transmission and dry season) from an SP child and an age-matched SN child and was performed by an investigator blinded to the age and schistosoma status of the samples. Thawed PBMC were rested overnight at 37°C, 5% CO_2_ and washed. A portion of cells (2.0 × 10^6^) was removed to serve as negative (media) and positive [stimulation with 10 µg/mL *Staphylococcus* enterotoxin B (SEB); Sigma, St. Louis, MO, USA] controls. The remaining PBMC were split into two aliquots consisting of ~1.0 × 10^6^ cells each and stimulated with a malaria antigen pool [consisting of AMA1 (29) and Merozoite Surface Protein 1 (MSP1_42_)] (30), and *S. haematobium* antigen pools [soluble egg antigen and soluble worm antigen protein (SWAP)]. Antigen stimulation for the assay was optimized at 5 μg/mL/antigen. The malaria antigens chosen represent two vaccine candidates being tested at the same field site in Mali, while the *S. haematobium* antigens are commonly used in schistosoma research. The 3D7 stain of malaria is well documented at the Malian site and strain-specific protection against an AMA1 vaccine has been established (31). AMA1 has been shown to elicit cell-mediated immunity (32). Using an optimized protocol (33), all cells were stimulated for 2 h before protein transport was blocked by adding 0.5 μL/tube GolgiPlug (BD Pharmingen) followed by overnight incubation. The secondary analysis examined PBMC stimulated with AMA1 (5 µg/mL) alone and processed in an identical fashion.

### Flow Cytometry Staining and Analysis

#### Primary Panel

Peripheral blood mononuclear cells were stained with fluorochrome-labeled monoclonal mouse anti-human antibodies (mAb) against surface antigens (CD3-Energy Coupled Dye (ECD, Beckman Coulter, clone UCHT1), CD4-Pacific Orange (BD Biosciences, clone SK3), CD8-FITC (BD Biosciences, clone HIT8a), CD19 (Invitrogen, clone SJ25-C1)/CD14 Invitrogen, clone TüK4)/Vivid-Pacific Blue, CD45RA Quantum Dots (Qdot) 655 (clone 5H3), and CD62L APC-EF780 (eBiosciences, clone DREG-56), followed by fixation/permeabilization by using cytofix/cytoperm solution (eBiosciences) and intracellular staining with mAb to IFN-γ-APC (BD Biosciences, clone B27), IL-2-phycoerythrin (PE)-Cy7 (BD Biosciences, clone MQ1-17H12), TNF Alexa Flour 700 (BD Biosciences, clone Mab11), IL-17A PerCP-Cy5.5 (eBiosciences, clone eBio64DEC17), and CD69-PE (eBiosciences, clone FN50).

#### Secondary Panel

A subset of unpaired PBMC (from the transmission season) were stained with CD3-ECD, CD4-APC-Cy7 (BD Biosciences, clone RPA-T4), CD8-PE-Cy7, CD19/CD14-PacBlue, biotinylated CD45RA (BD Biosciences, clone HI100) PacOrange, CD62L PE-Cy5 (BD Biosciences, clone DREG-56), IFN-γ-PE (BD Biosciences, clone B27), IL-4 Alexa488 (BioLegends, clone M8D4-8), and IL-10 APC (BioLegends, clone JES3-19F1) mAb as described above.

Cells were resuspended in 1% formaldehyde fixation buffer and analyzed using a BD LSR II SORP 4-laser flow cytometer. PBMC from healthy subjects were used as internal controls in the experiments. A total of 244,000–1,000,000 events (mean ~600,000) in the forward and side scatter (FS/SS) lymphocyte gate were collected per sample. List-mode data files were analyzed using WinList 7.1 3D (Verity Software House, Topsham, ME, USA). An amine reactive dye (ViViD, Invitrogen, OR, USA) was used as a dead cell discriminator and B lymphocytes (CD19^+^) and macrophages/monocytes (CD14^+^) were excluded from analysis. Doublets/aggregates were subtracted from analysis and gate placement determined with the aid of Fluorescence Minus One controls. Specimens were included in the analysis if (1) the cell viability was >80% after thawing and (2) cells were shown to be functionally active as determined by the production of IFN-γ by at least 0.2% CD3^+^ cells after stimulation with SEB. A response was considered specific if (1) the differential in the number of positive events in the stimulant pool compared to the media control was significantly increased by χ^2^ analyses; and (2) the net percentage of cytokine-producing cells was ≥0.1% in stimulant pool as compared to the media control. A response was considered positive if the production of one or more cytokines, meeting the pre-defined criteria, was measured in response to antigen stimulation of PBMC.

#### Statistics

Statistical analysis was performed on GraphPad Prism 5 (Graphpad Software, Inc., San Diego, CA, USA), and demographic and immunologic data were stratified and evaluated by age group (age 4–8 and 9–14 years). Student *t*-test (two-tailed) or Mann–Whitney *U* test were used to compare continuous and/or nonparametric data and χ^2^ analysis, using Mantel–Haenszel or Fisher Exact (two-tailed) as appropriate, was performed for categorical data. Cellular multifunctionality was assessed using the WinList 3D 7.1 FCOM to enumerate phenotypes. Spearman rank correlation coefficient was calculated utilizing GraphPad Prism 5. A significance level of *P* < 0.05 was considered statistically significant.

## Results

### Sample Population

#### Primary Dataset

Samples from children with >*10* × *10^6^ PBMC/time point* (*n* = 48, mean age 7.9 years, range 4–14 years) were thawed and examined. Evaluative data was available from 24 SP children (mean egg count 58 eggs/10 mL urine) and 24 SN children, all but two of who developed 1–4 malaria episodes during the transmission season (Table 1). One SP child who remained malaria-free was age-matched to an SN child who likewise, remained malaria-free over the course of the study period. Samples were excluded if the viability or thawed quantity of PBMC was insufficient (*n* = 3 time points). SP children had a statistically longer time to first clinical malaria infection and a trend toward a reduced geometric mean parasite density at the time of malaria infection but experienced similar numbers of malaria episodes over the course of a single transmission season (Table 1). There was no age-related difference in the character of the malaria infection in SP children. Children were deemed to be free of *S. haematobium* based upon 2–3 negative urine examinations and 1–2 stool examinations coupled with follow-up studies performed ~9 months later prior to the dry season follow-up. We have previously demonstrated similar prevalence of soil transmitted helminth infections and hemoglobinopathies (hemoglobin S and C) in these age-matched populations (3).

**Table 1:** Demographics.

Category	SP Mal (*n* = 24)	SN Mal (*n* = 24)	*P* value
Mean age (range)	7.8 (4–14)	8.0 (4–14)	0.73
Female (%)	9 (37.5)	15 (62.5)	0.09
Eggs (range)^a^	57.7 (2–466)	0	n/a
Malaria episodes^b^ (range)	2.19 (1–4)	2.23 (1–4)	0.90
Days to first malaria^b^ episode (range)	40.2 (49–113)	26.8 (3–40)	**0.006**
Parasitemia^b^^,^^c^ (range)	3,803 (750–90,000)	10,284 (1,025–105,000)	0.15

*Demographic characteristics at enrollment and features of subsequent *Plasmodium falciparum* malaria infections of *Schistosoma haematobium*-positive (SP Mal) and age-matched *S. haematobium*-negative (SN Mal) Malian children contributing peripheral blood mononuclear cells for primary immunologic analysis*.

*^a^Urinary egg excretion detected in 10 mL of filtered morning (10:00 a.m. to 2:00 p.m.) urine*.

*^b^Excluding two children (one SP and one SN) who remained malaria free during study follow-up*.

*^c^Geometric mean parasite density per cubic millimeter*.

*Bold indicates significant value with **P ≤** 0.05*.

### Intracellular Cytokine Expression to Antigenic Stimulation

#### Malaria Antigen

**Figure 1:**
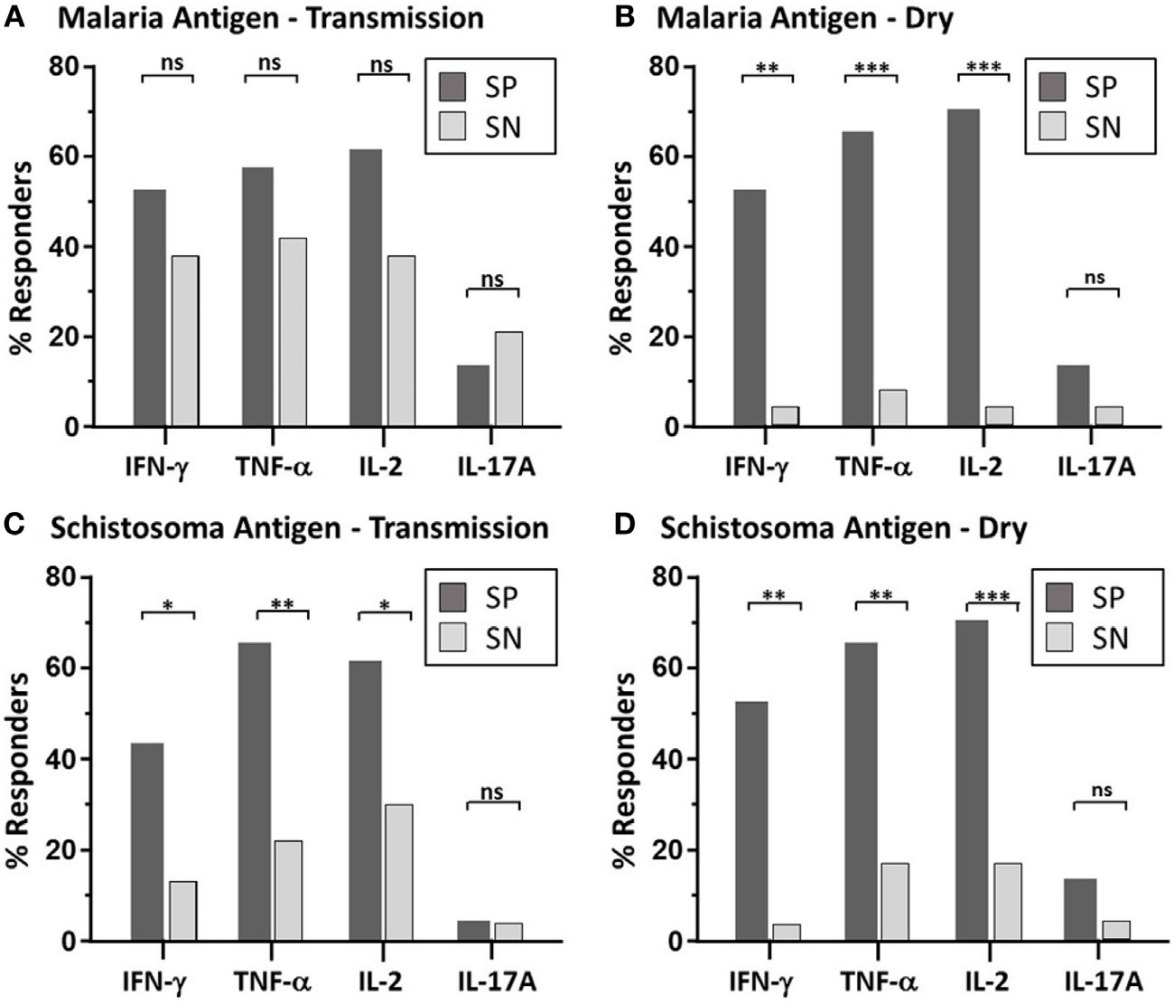
Maintenance of parasite-specific memory response—depicted is the bar graph representation of the percent of responders with significant memory response, as defined by CD4 T cell-derived expression to each individual cytokine (IFN-γ, TNF-α, IL2, and IL-17A), after malaria antigen pool (apical membrane antigen 1 and merozoite surface protein 1) **(A,B)** or Sh antigen pool (soluble egg antigen and soluble worm antigen protein) **(C,D)** stimulation. Peripheral blood mononuclear cells acquired from schistosoma-positive (SP: dark bar) or -negative (SN: light bar) Malian children aged 4–14 years acquired at the time of acute malaria during the transmission season and again, 6 months later, during the dry season were stimulated with parasite antigen and results were measured by flow cytometry after gating on CD19^−^CD14^−^CD3^+^CD8^−^CD4^+^ T cells after live/dead discrimination. χ^2^ results using Mantel–Haenszel analysis was performed between SP vs. SN children with statistically significant quantities of cytokine production after antigen stimulation. *P* value significance set at *<0.05; **<0.001; ***<0.0001. Not significant, ns. The dot plot depiction of the individual cytokine net percentage of response is depicted in Figure S2 in Supplementary Material.

Paired PBMC samples obtained at two time points (malaria transmission (wet) and dry season) were examined *via* multiparameter flow cytometry (Figure S1 in Supplementary Material). Intracellular cytokine production (IFN-γ, TNFα, IL2, and IL17A) from PBMC stimulated with pooled malaria and schistosoma antigens was measured (Table 2; Figure S2 in Supplementary Material) in 24 SP Mal and 24 SN Mal children (*n* = 48). The majority of cytokine-producing cells were found to derive from CD3^+^CD19^−^CD4^+^CD8^−^ T cells with a minority (<0.03%) observed from CD8^+^ T cells. The amount of cytokine measured after malaria antigen stimulation was low, but significant, with a geometric mean value of less than 0.2%. More SP children [15/23 (65%)] had detectable cytokine expression (one or more cytokines) than SN children [12/24 (50%); *P* = 0.29, Mantel–Haenszel χ^2^ analysis], during the malaria transmission season at the time of their malaria episode (Figure 1A); however, little difference was noted between the two groups in terms of the geometric mean percent of that cytokine response (Figure S2 in Supplementary Material). A small minority of children [3/23 (13%) SP and 5/24 (21%) SN children] had demonstrable IL-17A cytokine production at the time of an active malaria infection. Contrary to previous immunologic results (14, 15), we found no significant difference in cytokine production to antigen stimulation when stratified by age (Spearman rank correlation coefficient, ρ = −0.17, *P* = 0.26). When paired dry season samples were analyzed, fewer SN children [7/23 (30.4%)] had a recall response to malaria antigen (defined as one or more cytokine response) as compared to SP children, who not only retained the low-level memory response to malaria antigen but had a higher number of responders [16/23 (70%); *P* = 0.009, OR = 5.01, 95% confidence interval (CI) (1.27–22.77), Mantel–Haenszel χ^2^ analysis] Individual cytokines results demonstrate statistically significant results for IFN-γ, TNF-α, and IL-2 (Figure 1B). The overall amount of secreted cytokine (IFN-γ, TNF-α, IL-2, or IL-17A) remained statistically similar to that measured during the transmission season but without high responding outliers (Table 2; Figure S2 in Supplementary Material). Eleven of the 16 SP children with demonstrable malaria antigen recognition during the dry season had detectable cytokine production at the earlier, transmission season time point. Minimal detectable IL-17A was measured at the dry season timepoint, suggesting that this cytokine may be part of the inflammatory response mounted to acute infection but does not play a significant role in memory response post-transmission season. Malaria-naïve U.S. adult controls had no detectable increase of intracellular cytokines to either antigen stimulant.

**Table 2:** Intracellular cytokine expression to antigen stimulation.

Season	Cohort	Ag^a^	No. (IFNγ)^b^ (range)	*P*^c^	No. (TNFα) (range)	P^c^	No. (IL-2) (range)	*P*^c^	No. (IL-17A) (range)	*P*^c^
Wet^d^	SP	Pf	12/23 (0.22) (0.1–1.09)	0.32	13/23 (0.39) (0.1–1.8)	0.31	14/23 (0.32) (0.1–1.7)	0.11	3/23 (0.37) (0.1–0.91)	ns
	SN^c^	Pf	9/24 (0.22) (0.1–0.86)		10/24 (0.19) (0.1–0.57)		9/24 (0.29) (0.1–1.04)		5/24 (0.25) (0.1–0.71)	
	SP	Sh	12/23 (0.25) (0.1–0.69)	**0.0003**	15/23 (0.28) (0.1–0.75)	**<0.0001**	16/23 (0.23) (0.1–0.72)	**<0.0001**	3/23 (0.09) (0.1)	0.28
	SN	Sh	1/24 (0.15) (n/a)		2/24 (0.36) (0.15–0.55)		1/24 (0.50) (n/a)		1/24 (0.1) (n/a)	

Dry^d^	SP	Pf	10/23 (0.24) (0.1–1.08)	0.02	15/23 (0.29) (0.1–1.89)	**0.003**	14/23 (0.29) (0.1–1.7)	**0.04**	1/23 (0.10) (n/a)	ns
	SN	Pf	3/23 (0.12) (0.1–0.15)		5/23 (0.18) (0.11–0.34)		7/23 (0.15) (0.1–0.32)		1/23 (0.13) (n/a)	
	SP	Sh	12/23 (0.25) (0.1–0.57)	**0.0004**	15/23 (0.33) (0.1–0.81)	**0.001**	18/23 (0.26) (0.1–1.00)	**<0.0001**	4/23 (0.17) (0.1–0.31)	0.16
	SN	Sh	1/23 (0.25) (n/a)		4/23 (0.40) (0.15–0.72)		4/23 (0.44) (0.19–0.95)		1/23 (0.17) (n/a)	

*Results of the intracellular cytokine expression after antigenic stimulation of peripheral blood mononuclear cells (PBMC) acquired from age-matched children with [schistosomiasis-positive (SP)] or without Schistosoma Haematobium [schistosomiasis-negative (SN)] during the malaria transmission (wet) season at the time of a malaria episode and again during a dry season follow-up visit. The results represent those individuals with statistically significant increases in the percentage of cytokine expressed compared to media controls after PBMC stimulation and the denominator represents the total number of individual PBMC samples analyzed. The results are depicted in graphical form in Figure 1*.

*^a^PBMC were stimulated with a malaria antigen pool (apical membrane antigen 1 and merozoite surface protein 1) or with an *S. haematobium* pool (soluble egg antigen and soluble worm antigen protein)*.

*^b^Number (No.) of individuals with statistically significant cytokine expression, the geometric mean of the net% of cytokine detected (stimulant minus media control) in positive individuals, and the range measured*.

*^c^χ^2^ analysis, using Mantel–Haenszel analysis, was performed between SP vs. SN children with statistically significant quantities of cytokine production after antigen stimulation. P value significance set at <0.05. Not significant, ns*.

*^d^Three experiments were excluded due to insufficient or poor viability cell quantities to allow proper interpretation of data*.

*Bold indicates significant value with **P ≤** 0.05*.

#### *S. haematobium* (Sh) Antigen

Paired PBMC were stimulated with Sh antigen as described above. Significant production of at least one cytokine was measured after Sh antigen stimulation in the majority of SP children [17/23 (74%)] compared to 2/24 (8.3%) of SN children [*P* = < 0.0001, 95% CI (4.69–271), χ^2^ analysis]. As noted with the malaria antigen, the net percentage of cytokine expression, in those that had a demonstrable cytokine response, was not significantly different between groups (Table 2; Figure S2 in Supplementary Material), but the number of children with antigen-specific cytokine production was quite different (Figure 1). At the dry season time point, and prior to administration of praziquantel, 18/23 (78.3%) SP children had detectable PBMC cytokine production compared to 4/23 (17.4%) of SN children [*P* = < 0.0001, 95% CI (3.3–102), χ^2^ analysis]. Of the SN children with detectable response to Sh antigen, none had evidence of urinary egg secretion at follow-up. One SN child demonstrated a particularly robust response to antigen at both the wet and dry season time point, without detectable egg secretion, suggesting that this individual may have been previously exposed or developed inherent or putative resistance to urinary schistosomiasis (34). The remaining three children had modest responses at a single time point, which may also represent a sub-clinical infection or a false positive response; however, malaria-naïve U.S. adult controls had no detectable increase of intracellular cytokines to Sh antigen.

#### Multifunctional T Cell and Memory Subpopulations Analysis

**Figure 2:**
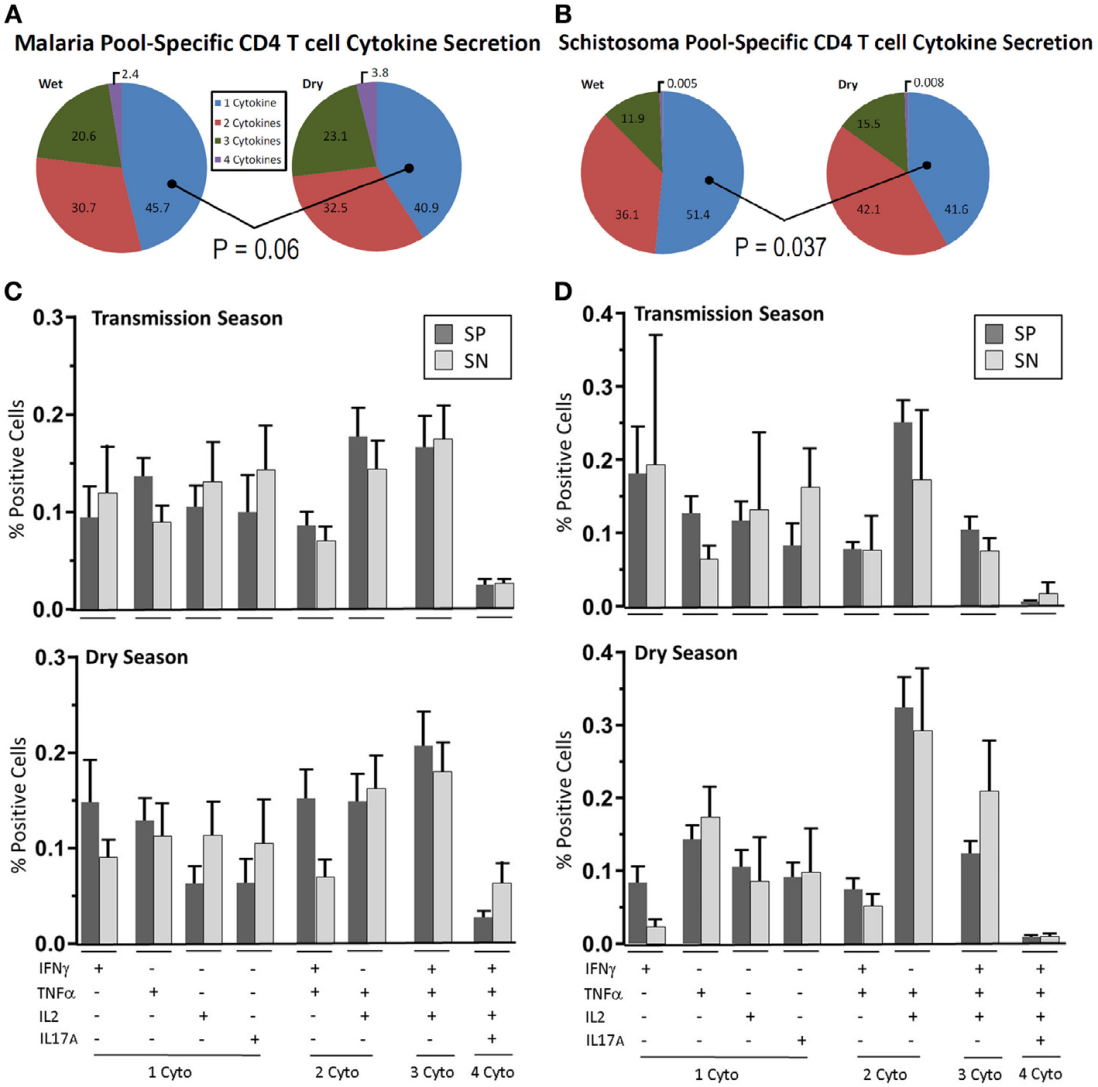
Parasite-specific multifunctional cytokine expression over time. Depicted are pie-charts **(A,B)** showing the multifunctional nature, expressed as geometric mean of singlet, doublet, triplet, and quadruplet cytokine (IFN-γ, TNF-α, IL2, and IL-17A) combinations secreted from CD4^+^ T cells after peripheral blood mononuclear cells (PBMC) stimulation with either **(A)** malaria antigen pool (apical membrane antigen 1 and merozoite surface protein 1) or **(B)** schistosoma antigen pool (soluble egg antigen and soluble worm antigen protein). PBMC were acquired from schistosoma-negative (SN) or -positive (SP) Malian children aged 4–14 years at the time of acute malaria during the transmission season and again, 6 months later, during the dry season. The most prevalent cytokine combinations expressed to malaria antigen stimulation **(C)** or schistosoma antigen stimulation **(D)** are depicted for each group (SP—dark bar, SN—light bar) during the transmission season and dry season. Results are depicted as the mean percentage of positive cells with SE. The dot plot depiction of individual cytokine net percentage of response is depicted in Figure S3 in Supplementary Material.

In PBMC found to have antigen recognition to malaria or schistosoma antigen, we measured multifunctional T cell responses (secreting two or more cytokines at a single cell level) at the time of malaria infection and in post-season follow-up (dry season). We also examined major memory subpopulations producing cytokines in PBMC specimens from SP and SN children (Figures 2A,B).

#### Malaria

During the acute malaria episode, 45.7% of cytokine-secreting CD4^+^ T cells expressed a single cytokine after stimulation with malaria antigen with the remainder expressing more than one cytokine (Figure 2A). The distribution and mean values of the most prevalent combinations of cytokine secretion are depicted in Figure 2C (raw data are depicted in Figure S3 in Supplementary Material). Over the duration of follow-up, single cytokine expression at a cellular level fell to 40.9% (*P* = 0.06) of the total number of cytokine-producing cells.

Of the multifunctional CD4^+^ T cells measured, 30.7% were noted to be double-positive and 20.8% were triple-positive. The majority of triple-positive (81.7%) were IFN-γ^+^TNF-α^+^IL2^+^-expressing at the time of an acute malaria episode. A minor population of quadruple-positive CD4^+^ T cells was also measured (2.4%). This increased slightly to 32.5% double-positive, 23.1% triple-positive and 3.8% quadruple positive at the dry season follow-up with the majority (86.6%) of triple-positive again being IFN-γ^+^TNF-α^+^IL2^+^-expressing. The percentage rise of multifunctional cytokine secretion over time was incremental but persistent across all combinations of cytokine but did not reach statistical significance in any one particular cytokine combination (Figures 2C,D). IL-17A production was measured in the acute malaria stage with expression being significantly reduced at the dry season follow-up. An inverse relationship was noted between IFN-γ and IL-17A. In those children with elevated CD4^+^ T cell IFN-γ expression to antigen stimulation, low IL-17A levels were measured and vice versa (Spearman rank sum = 0.2515, ρ = 0.04).

**Figure 3:**
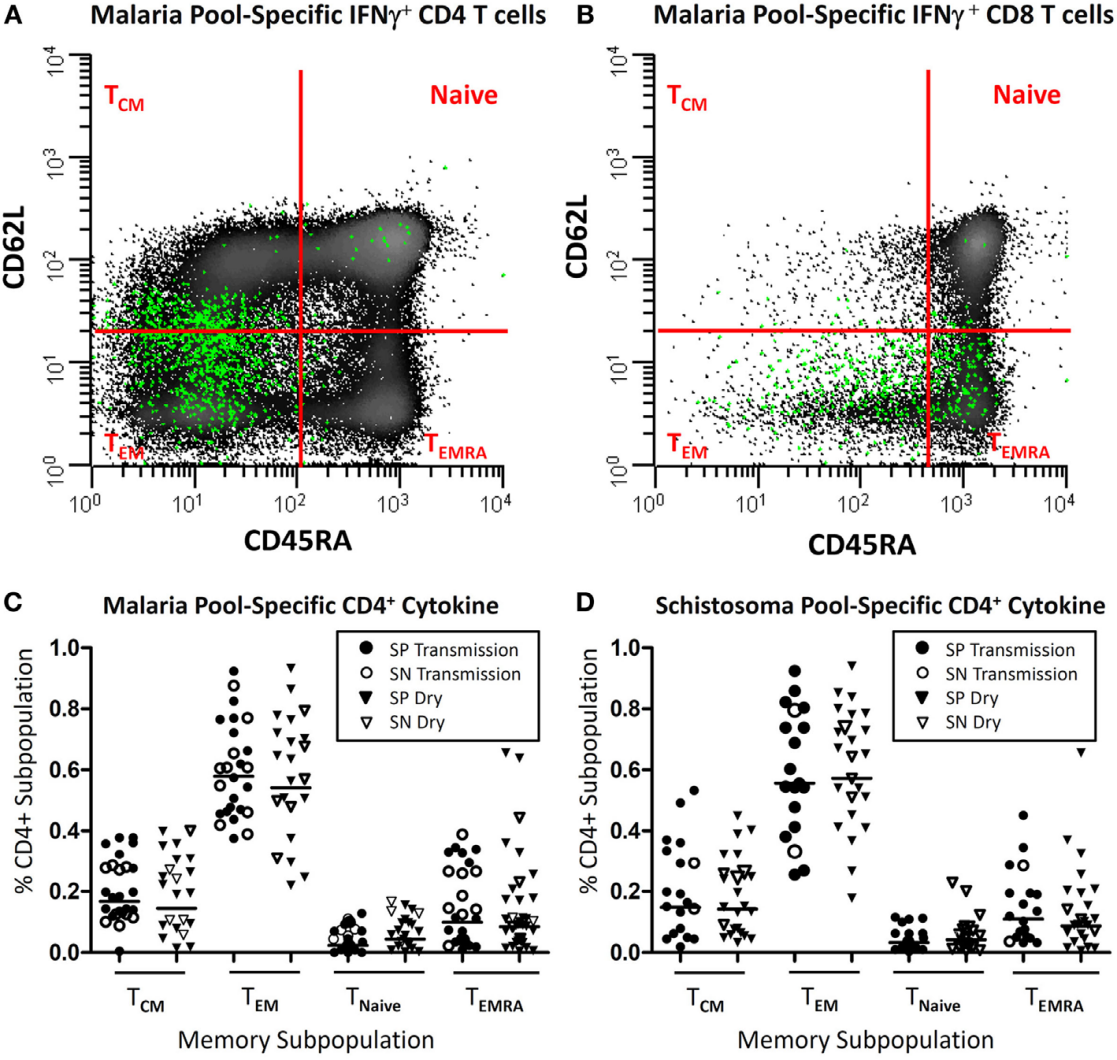
Parasite-specific memory subpopulations. Depicted is a representative example **(A,B)** of where T cells expressing intracellular IFN-γ (green) after antigen stimulation (malaria pool consisting of apical membrane antigen 1 and merozoite surface protein 1) distribute after being back gated onto the total population CD3^+^CD19^−^CD14^−^ cells following live/dead discrimination and stratified as CD4^+^ **(A)** or CD8^+^ **(B)** from a child with acute *Plasmodium falciparum* infection and further stratified by memory subpopulation utilizing CD45RA and CD62L surface markers. Panels **(C,D)** depict the distribution of cytokine-secreting (either IFN-γ, TNF-α, IL2, and/or IL17A), malaria or schistosoma-specific CD4^+^ T cells, stratified by memory subpopulation, in children with (SP) or without *Schistosoma haematobium* (SN), during the transmission season and at a later convalescent time point (dry season).

Upon examination of the memory subpopulation from which cytokine-secreting cells derived, it was noted that the majority of inflammatory cytokines (range 55.5–61.4% geometric mean per cohort) derived from CD45RA^−^CD62L^−^ effector memory T cells (T_EM_). If there was a detectable memory response, there was little variation depending upon the time point (transmission vs. dry season) or the study cohort (SP vs. SN). Results of SP and SN groups at both time points are depicted (Figure 3). CD45RA^−^CD62L^+^ central memory T cells (T_CM_) accounted for 13.9–16.8% of cytokine secretion. A minority of cytokine derived from CD45RA^+^ effector memory T cells (T_EMRA_) (7.7–14%) population and naïve T cells (2.1–4.7%).

#### *Schistosoma* *haematobium*

Of the children with detectable Sh antigen recognition, 51.4% of all cytokine-secreting cells expressed a single cytokine while the remainder expressed two or more cytokines (Figures 2B–D). The most prevalent cytokine expressed by cytokine-secreting cells was IFN-γ expressed by 18.3% of these cells at the time of the acute malaria episode. This value fell to 7.2% by the dry season convalescent appointment (*P* = 0.06). TNF-α production remained relatively stable comprising 12.2% of single cytokine-producing cells at the time of acute malaria and 14.9% during the dry season follow-up.

Among the multifunctional CD4^+^ T cells measured, 36.1% were noted to be double-positive, 11.9% were triple-positive, and a small percentage were quadruple-positive (0.5%) reflective of the low prevalence of IL-17A measured to *S. haematobium* infection. The majority of triple-positive [10.5/11.9 (88.2%)] were IFN-γ^+^TNFα^+^IL2^+^-expressing at the time of an acute malaria episode. Over time an incremental but persistent increase in multifunctional cell cytokine production was noted across all cytokine combinations (similar to results reported for malaria antigen stimulation, Figure 2B). At the dry season follow-up, the percentage of single-cytokine-producing cells fell to 41.6% (transmission season vs. dry season, *P* = 0.037, two-tailed Mann–Whitney Rank Sum), 42.1% double-positive (*P* = 0.27), 15.5% triple-positive (*P* = 0.38) and 0.8% (*P* = 0.58) quadruple positive. Raw data can be found in Figure S3 in Supplementary Material.

There was little variation in memory subpopulation in which cytokine production was observed, between seasons or study cohorts. SP and SN results were combined for analysis and reported as geometric mean values (Figure 3). The majority of cytokine (51.4–62.9%) derived from T_EM_. T_CM_ accounted for 14–22% of cytokine secretion with slightly more T_CM_ noted in the small subset of SN compared to SP population (13.5 vs. 22.5%, *P* = 0.20). A minority of cytokine was detected from cells of T_EMRA_ (5–11%) population and naïve T cells (2–4%).

#### Secondary Subset Analysis

**Figure 4:**
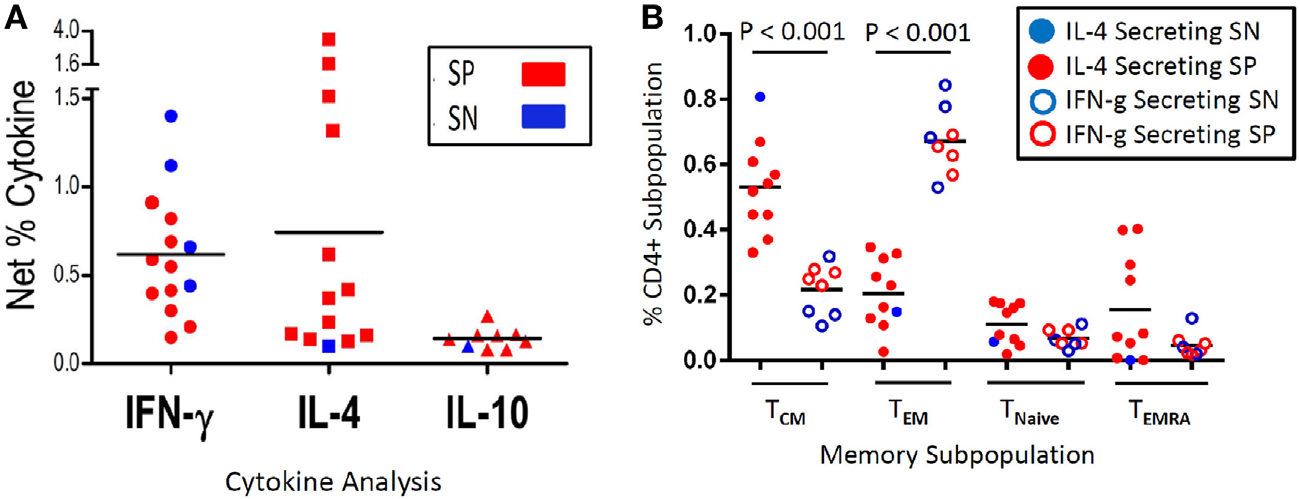
Malaria antigen-specific cytokine expression and corresponding memory subpopulation. Depicted is the net percentage of CD4^+^ T cell-derived IFN-γ, IL-4, or IL-10 cytokine secreted from peripheral blood mononuclear cells (PBMC) acquired from schistosoma-positive (SP) or -negative (SN) Malian children with evidence of antigen recognition after stimulation with malaria apical membrane antigen 1 antigen **(A)**. Cells were gated on CD19^−^CD14^−^CD3^+^CD8^−^CD4^+^ after live/dead discrimination. Panel **(B)** depicts the memory subpopulation distribution from those PBMC that predominantly secreted IL-4 (a representative T_H_2 cytokine) (left column, solid dots, *N* = 9 SP, and 1 SN) or IFN-γ (a representative inflammatory T_H_1 cytokine) (right column, open dots, *N* = 4 SN and 4 SP) as depicted in Panel A (IL-10 not depicted). PBMC that expressed both cytokines (*N* = 7 SP and 0 SN) were categorized based on the dominant cytokine (defined as >50%) expressed. The memory subpopulations from one volunteer that secreted equal amounts of IL-4 and IFN-γ was excluded **(B)**. Memory subpopulations were defined as T central memory (T_CM_—CD62L^+^CD45RA^−^), T effector memory (T_EM_—CD62L^−^CD45RA^−^), Naïve T cells (T_Naive_—CD62L^+^CD45RA^+^), or T effector memory RA^+^ (T_EMRA_—CD62L^−^CD45RA^+^). The mean value is represented as a horizontal bar and statistical significance set at *P* < 0.05.

A secondary experiment utilized unmatched transmission season samples from Malian children from this same study (*n* = 63, mean age 8.3 years, range 4–13 years), which were thawed and examined as part of a previously unpublished data set. Evaluative data were available from 30 SP children and 30 SN children who developed between 1 and 5 malaria episodes during a single transmission season (Table S1 in Supplementary Material). Among PBMC stimulated with malaria antigen AMA1, 4/30 (13.3%) SN vs. 10/30 (33%) SP expressed IFN-γ (*P* = 0.07, OR 2.5, CI: 0.71–9.99, χ^2^ analysis); 1/30 (3.3%) SN vs. 12/30 (40%) SP expressed IL-4 (*P* = 0.002, OR 11.7, CI: 1.8–266.8, Fisher Exact); and 1/30 (3.3%) SN vs. 7/30 (23.3%) SP expressed IL-10 (*P* = 0.05, OR 6.8, CI: 0.97–163.8, Fisher Exact) (Figure 4A). While PBMC from eight SP children expressed both IFN-γ and IL-4, in all but one case, one cytokine was predominantly expressed (defined as >50%). One child had similar amounts of IL-4 and IFN-γ cytokine expression. We then examined the CD45RA^−^ memory subpopulations, divided into CD62L^−^ T effector memory (T_EM_) and CD62L^+^ T central memory (T_CM_), relative to the cytokine expression profile. Of those children that had an inflammatory expression profile characterized by IFN-γ expression, the percentage of T_EM_ predominated as compared to those children with a dominant IL-4 expression profile (mean T_EM_: 66.9 vs. 20.4%, *P* < 0.001). Additionally, those children with an IL-4 expression profile (predominantly SP children) had a higher percentage of T_CM_ as compared to those children with a dominant IFN-γ expression (mean T_CM_: 53 vs. 22.2%, *P* < 0.001) (Figure 4B). There was no age-related correlation with cytokine expression between SP and SN children and no change in T naïve or effector memory RA^+^ (EMRA) populations. However, when SP children were stratified between those expressing an IL-4 profile (*n* = 9) and those with an IFN-γ profile (*n* = 4), children expressing IFN-γ were significantly older (7.4 vs. 13 years, *P* < 0.001).

## Discussion

Very little is known about the immunologic perturbations that one parasitic infection inflicts upon the immune response to another infection when they co-occur in the human host. We have demonstrated an age-specific reduction in *P. falciparum* malaria acquisition among Malian children, aged ≤9 years, infected with *S. haematobium* over the course of a single transmission season (3). In this study, we have measured detectable CD4^+^ T cell memory response against both malaria antigens and *Sh* antigens and demonstrated multifunctional cellular responses against both antigens during an active malaria transmission season. The responses to the malaria antigens were similar among age-matched children with and without *Sh*. Lower CD8^+^ T cell response was noted against either antigen in both populations. At a convalescent, dry season time point after malaria transmission ceased, children with chronic *Sh* infection appeared to retain their memory responses to malaria antigen whereas uninfected children’s memory response waned considerably (70 vs. 30%). SP children also had similar memory responses to Sh antigens at both time points. The quality of these cellular responses, as measured by cellular multifunctionality, increased over the 6- to 9-month time interval with elevated dual, triple, and quadruple cytokine-secreting cells to malaria and Sh antigens at the convalescent time point.

The role of CD4^+^ T cells is well established in malaria. However, the presence of CD4^+^ T cell memory response in chronically infected individuals with schistosomiasis and the kinetics of these cells is poorly understood. While CD4^+^ T cell memory cells are thought to provide protection against re-infection by schistosomes, the prevailing belief is that chronic helminth infection leads to suppression of the CD4 T cell memory compartment. Moreover, as we have demonstrated, helminth infections can modulate the host adaptive response against itself and other “bystander” antigens (12, 13, 15). Mutapi et al. reported that the CD4^+^ T_EM_ populations were decreased in schistosoma-infected individuals compared to uninfected individuals and that the CD4^+^ T cell memory population paradoxically decreased after treatment of the helminth-infected individuals (35). We examined cell populations after antigen stimulation; a notable design difference. This analysis was performed in a subset of children utilizing a flow panel that incorporated the measurement of IL-4 production. We demonstrated enhanced CD4^+^ T_EM_ in PBMC from Malian children who demonstrated antigen recognition of malaria proteins, which appeared predominantly in children with an inflammatory expression profile characterized by IFN-γ secretion. We observed this expression profile in most SN and some SP children. Of importance, those children with an IL-4 expression dominance (representative of a T_H_2 cytokine profile and chronic schistosomiasis) had significantly elevated CD4^+^ T_CM_ populations. Helminth-infected children comprised the majority of those with a dominant IL-4 expression profile, with only one uninfected exception; however, a subset of SP children demonstrated an inflammatory-dominant profile. The subset of SP children with an inflammatory-dominant profile were older than those with an IL-4 dominant profile (13 vs 7.4 years, *P* < 0.0001). This fact would argue against the inflammatory-dominant profile in SP children being due to an acute Sh infection as Malian children would have been exposed at a much younger age. This is also consistent with the finding that IFN-γ production in schistomiasis-infected individuals is associated with acquisition of anti-helminth immunity (36). Of note, four of the IL-4 producing SP children had detectable IL-10 production as well, which has been associated with helminth-induced anergy and hyporesponsiveness as well as downregulatory responses (37, 38).

While much of the protective immune response to malaria is thought to be due to liver tissue-resident CD8 T cells, IL-4-secreting CD4^+^ T cells are thought to be critical for mediating CD8 T cell response to malaria liver antigens (39). Additionally, CD8 T cell memory response are dependent upon IL-4 and IL-4 receptor interaction at the level of the liver in the murine model (40). It is well established that cell-mediated immunity to malaria rapidly wanes in children and that acquisition of immunity requires repetitive exposure to malaria. Therefore, it is remarkable that the children with concomitant Sh appeared to retain their memory response to malaria antigen at a time point 6–9 months after their malaria exposure. This was not seen in Sh-negative, age-matched children followed over the same malaria transmission season. It is also possible that the effects simply reflect a previously undescribed cross-reaction between antigens but we have no evidence to support or refute this possibility. Our results provide the first evidence in humans, albeit indirect, that an immunologic milieu rich in IL-4, such as seen in a helminth infection, may contribute to enhanced T cell memory against a bystander antigens, such as those encountered in malaria. Moreover, while numbers were small, older SP children had an inflammatory expression profile analogous to uninfected children. This might provide an immunologic explanation for our field findings that children aged 9 years or less, and infected with Sh, had modest protection against the acquisition of *P. falciparum* malaria compared to age-matched uninfected children or older children with Sh.

## Ethics Statement

This trial was carried out in accordance with the recommendations of the University of Bamako and the University of Maryland Institutional Review Boards (IRBs) with written consent obtained from all legal guardians. Village permission to conduct research was obtained from village chiefs, government officials, and traditional healers prior to study initiation. Individual written informed consent was obtained from the parent or legal guardian of each child prior to screening and enrollment in accordance with the Declaration of Helsinki. All children displaying gross hematuria or symptoms of genitourinary pathology were treated with praziquantel (40 mg/kg) therapy and discharged from the study.

## Author Contributions

KL obtained funding; KL, OD, CP, and MS participated in study design; KL, AD, CA, ID, CP, and OD participated in study conduct and regulatory review. KL and MS participated in immunologic assays and interpretation; KL, CP, and MS participated in writing of manuscript; all authors participated in editing of manuscript.

## Conflict of Interest Statement

The authors declare that the research was conducted in the absence of any commercial or financial relationships that could be construed as a potential conflict of interest.

## Supplementary Material

Figure S1 Gating strategy. Gating strategy for CD4^+^ memory response depicting **(A)** representative IFN-γ cytokine expression in response to malaria antigen stimulation from the central memory (CD62L^+^CD45RA^−^), naïve (CD62L^+^CD45RA^+^), effector memory (CD62L^−^CD45RA^−^), and EMRA (CD62L^−^CD45RA^+^) subpopulations. The media control and the malaria antigen-stimulated peripheral blood mononuclear cells (PBMC) from schistosoma-positive children and gated on CD19^−^CD14^−^CD8^−^CD4^+^ T cells after live/dead discrimination **(B–G)**. Panels **(B–E)** depict CD69^+^ cytokine expression. Panels **(F,G)** are from an alternate panel and represent the total cytokine expressed from CD4^+^ T cells.

Figure S2 Intracellular cytokine expression to antigen stimulation. Depicted is the dot plot representation of the overall CD4^+^ T cell immune response, expressed as produced cytokine (in net%), to either malaria antigen pool (apical membrane antigen 1 and merozoite surface protein 1) **(A,B)** or Sh antigen pool (soluble egg antigen and soluble worm antigen protein) **(C,D)**. Peripheral blood mononuclear cells (PBMC) acquired from schistosoma-negative [schistosomiasis-negative (SN)—hollow dot] or -positive [schistosomiasis-positive (SP)—solid dot] Malian children aged 4–14 years at the time of acute malaria during the transmission season and again, 6 months later, during the dry season. PBMC were stimulated with parasite antigen and results were measured by flow cytometry after gating on CD19^−^CD14^−^CD3^+^CD4^+^CD8^−^ T cells after live/dead discrimination. Only children with positive responses (i.e., significant by χ^2^ analysis and >0.1% over background; as described in Section “Materials and Methods”) are shown. Horizontal bars represent the geometric mean values of IFN-γ, TNF-α, IL2, and IL-17A for SN and SP children combined.

Figure S3 Seasonal parasite-specific multifunctional cytokine expression. Depicted are the most prevalent cytokine combinations elicited after parasite antigen stimulation **(A,B)** in individual children [schistosomiasis-positive (SP)—solid, schistosomiasis-negative (SN)—hollow] stratified by number of cytokines expressed during the transmission season and dry season. Peripheral blood mononuclear cells acquired from schistosoma-negative (SN—hollow shapes) or -positive (SP—solid shapes) Malian children aged 4–14 years at the time of acute malaria during the transmission season and again, 6 months later, during the dry season were stimulated with either **(A)** malaria antigen pool (apical membrane antigen 1 and merozoite surface protein 1) or **(B)** schistosoma antigen pool (soluble egg antigen and soluble worm antigen protein).

## References

[1] World Malaria Report 2016. World Health Organization. (2016). Available from: http://www.who.int/malaria/publications/world-malaria-report-2016/report/en/

[2] World Health Organization. Schistosomiasis, Fact Sheet No. 115. (2017). Available from: http://www.who.int/mediacentre/factsheets/fs115/en/

[3] Lyke KE Dicko A Dabo A Sangare L Kone A Coulibaly D Association of *Schistosoma haematobium* infection with protection against acute *Plasmodium falciparum* malaria in Malian children. Am J Trop Med Hyg (2005) 73:1124–30. 10.4269/ajtmh.2005.73.1124 16354824 PMC2738948

[4] Briand V Watier L Le Hesran JY Garcia A Cot M. Coinfection with *Plasmodium falciparum* and *Schistosoma haematobium*: protective effect of schistosomiasis on malaria in Senegalese children? Am J Trop Med Hyg (2005) 72:702–7. 10.4269/ajtmh.2005.72.702 15964953

[5] Pearce EJ Caspar P Grzych JM Lewis FA Sher A. Downregulation of Th1 cytokine production accompanies induction of Th2 responses by a parasitic helminth, *Schistosoma mansoni*. J Exp Med (1991) 173:159–66. 10.1084/jem.173.1.159 1824635 PMC2118762

[6] Grzych JM Pearce E Cheever A Caulada ZA Caspar P Heiny S Egg deposition is the major stimulus for the production of Th2 cytokines in murine *Schistosomiasis mansoni*. J Immunol (1991) 146:1322–7. 1825109

[7] Baumgart M Tompkins F Leng J Hesse M. Naturally occurring CD4+Foxp3+ regulatory T cells are an essential, IL-10-independent part of the immunoregulatory network in *Schistosoma mansoni* egg-induced inflammation. J Immunol (2006) 176:5374–87. 10.4049/jimmunol.176.9.5374 16622005

[8] Lyke KE Dabo A Sangare L Arama C Daou M Diarra I Effects of concomitant *Schistosoma haematobium* infection on the serum cytokine levels elicited by acute *Plasmodium falciparum* malaria infection in Malian children. Infect Immun (2006) 74:5718–24. 10.1128/IAI.01822-05 16988248 PMC1594876

[9] Remoue F Diallo TO Angeli V Herve M De Clercq D Schacht AM Malaria co-infection in children influences antibody response to schistosome antigens and inflammatory markers associated with morbidity. Trans R Soc Trop Med Hyg (2003) 97:361–4. 10.1016/S0035-9203(03)90170-2 15228260

[10] Diallo TO Remoue F Schacht AM Charrier N Dompnier JP Pillet S Schistosomiasis co-infection in humans influences inflammatory markers in uncomplicated *Plasmodium falciparum* malaria. Parasite Immunol (2004) 26:365–9. 10.1111/j.0141-9838.2004.00719.x 15679634

[11] Diallo TO Remoue F Gaayeb L Schacht AM Charrier N De CD Schistosomiasis coinfection in children influences acquired immune response against *Plasmodium falciparum* malaria antigens. PLoS One (2010) 5:e12764. 10.1371/journal.pone.0012764 20856680 PMC2939900

[12] Hartgers FC Yazdanbakhsh M. Co-infection of helminths and malaria: modulation of the immune responses to malaria. Parasite Immunol (2006) 28:497–506. 10.1111/j.1365-3024.2006.00901.x 16965285

[13] Hartgers FC Obeng BB Kruize YC Dijkhuis A McCall M Sauerwein RW Responses to malarial antigens are altered in helminth-infected children. J Infect Dis (2009) 199:1528–35. 10.1086/598687 19392626

[14] Lyke KE Wang A Dabo A Arama C Daou M Diarra I Antigen-specific B memory cell responses to *Plasmodium falciparum* malaria antigens and *Schistosoma haematobium* antigens in co-infected Malian children. PLoS One (2012) 7:e37868. 10.1371/journal.pone.0037868 22693628 PMC3367916

[15] Lyke KE Dabo A Arama C Daou M Diarra I Wang A Reduced T regulatory cell response during acute *Plasmodium falciparum* infection in Malian children co-infected with *Schistosoma haematobium*. PLoS One (2012) 7:e31647. 10.1371/journal.pone.0031647 22348117 PMC3279404

[16] Renia L Marussig MS Grillot D Pied S Corradin G Miltgen F In vitro activity of CD4+ and CD8+ T lymphocytes from mice immunized with a synthetic malaria peptide. Proc Natl Acad Sci U S A (1991) 88:7963–7. 10.1073/pnas.88.18.7963 1680235 PMC52425

[17] Schwenk RJ Richie TL. Protective immunity to pre-erythrocytic stage malaria. Trends Parasitol (2011) 27:306–14. 10.1016/j.pt.2011.02.002 21435951

[18] Darrah PA Patel DT De Luca PM Lindsay RW Davey DF Flynn BJ Multifunctional TH1 cells define a correlate of vaccine-mediated protection against *Leishmania major*. Nat Med (2007) 13:843–50. 10.1038/nm1592 17558415

[19] Seder RA Darrah PA Roederer M. T-cell quality in memory and protection: implications for vaccine design. Nat Rev Immunol (2008) 8:247–58. 10.1038/nri2274 18323851

[20] Wipasa J Elliott S Xu H Good MF. Immunity to asexual blood stage malaria and vaccine approaches. Immunol Cell Biol (2002) 80:401–14. 10.1046/j.1440-1711.2002.01107.x 12225376

[21] McGregor IA The development and maintenance of immunity to malaria in highly endemic areas. Clin Trop Med Commun Dis (1986) 1:1–29.

[22] Kullberg MC Pearce EJ Hieny SE Sher A Berzofsky JA. Infection with *Schistosoma mansoni* alters Th1/Th2 cytokine responses to a non-parasite antigen. J Immunol (1992) 148:3264–70. 1533656

[23] Elrefaei M El Sheikh N Kamal K Cao H. HCV-specific CD27-CD28- memory T cells are depleted in hepatitis C virus and *Schistosoma mansoni* co-infection. Immunology (2003) 110:513–8. 10.1111/j.1365-2567.2003.01769.x 14632650 PMC1783079

[24] Metenou S Dembele B Konate S Dolo H Coulibaly YI Diallo AA Filarial infection suppresses malaria-specific multifunctional Th1 and Th17 responses in malaria and filarial coinfections. J Immunol (2011) 186:4725–33. 10.4049/jimmunol.1003778 21411732 PMC3407819

[25] Coulibaly D Diallo DA Thera MA Dicko A Guindo AB Kone AK Impact of preseason treatment on incidence of falciparum malaria and parasite density at a site for testing malaria vaccines in Bandiagara, Mali. Am J Trop Med Hyg (2002) 67:604–10. 10.4269/ajtmh.2002.67.604 12518850

[26] De Clercq D Rollinson D Diarra A Sacko M Coulibaly G Landoure A Schistosomiasis in Dogon country, Mali: identification and prevalence of the species responsible for infection in the local community. Trans R Soc Trop Med Hyg (1994) 88:653–6. 10.1016/0035-9203(94)90212-7 7886759

[27] Corachan M Ruiz L Valls ME Gascon J Schistosomiasis and the Dogon country (Mali) [see comments]. Am J Trop Med Hyg (1992) 47:6–9. 10.4269/ajtmh.1992.47.6 1636885

[28] Lyke KE Burges RB Cissoko Y Sangare L Kone A Dao M HLA-A2 supertype-restricted cell-mediated immunity by peripheral blood mononuclear cells derived from Malian children with severe or uncomplicated *Plasmodium falciparum* malaria and healthy controls. Infect Immun (2005) 73:5799–808. 10.1128/IAI.73.9.5799-5808.2005 16113298 PMC1231120

[29] Dutta S Lalitha PV Ware LA Barbosa A Moch JK Vassell MA Purification, characterization, and immunogenicity of the refolded ectodomain of the *Plasmodium falciparum* apical membrane antigen 1 expressed in *Escherichia coli*. Infect Immun (2002) 70:3101–10. 10.1128/IAI.70.6.3101-3110.2002 12011004 PMC127972

[30] Angov E Aufiero BM Turgeon AM Van HM Ockenhouse CF Kester KE Development and pre-clinical analysis of a *Plasmodium falciparum* merozoite surface protein-1(42) malaria vaccine. Mol Biochem Parasitol (2003) 128:195–204. 10.1016/S0166-6851(03)00077-X 12742586

[31] Thera MA Doumbo OK Coulibaly D Laurens MB Ouattara A Kone AK A field trial to assess a blood-stage malaria vaccine. N Engl J Med (2011) 365:1004–13. 10.1056/NEJMoa1008115 21916638 PMC3242358

[32] Lyke KE Daou M Diarra I Kone A Kouriba B Thera MA Cell-mediated immunity elicited by the blood stage malaria vaccine apical membrane antigen 1 in Malian adults: results of a phase I randomized trial. Vaccine (2009) 27:2171–6. 10.1016/j.vaccine.2009.01.097 19356621 PMC2707027

[33] Salerno-Goncalves R Wahid R Sztein MB. Ex Vivo kinetics of early and long-term multifunctional human leukocyte antigen E-specific CD8+ cells in volunteers immunized with the Ty21a typhoid vaccine. Clin Vaccine Immunol (2010) 17:1305–14. 10.1128/CVI.00234-10 20660136 PMC2944457

[34] Oliveira RR Figueiredo JP Cardoso LS Jabar RL Souza RP Wells MT Factors associated with resistance to *Schistosoma mansoni* infection in an endemic area of Bahia, Brazil. Am J Trop Med Hyg (2012) 86:296–305. 10.4269/ajtmh.2012.11-0204 22302866 PMC3269284

[35] Nausch N Bourke CD Appleby LJ Rujeni N Lantz O Trottein F Proportions of CD4+ memory T cells are altered in individuals chronically infected with *Schistosoma haematobium*. Sci Rep (2012) 2:472. 10.1038/srep00472 22737405 PMC3382734

[36] Jankovic D Wynn TA Kullberg MC Hieny S Caspar P James S Optimal vaccination against *Schistosoma mansoni* requires the induction of both B cell- and IFN-gamma-dependent effector mechanisms. J Immunol (1999) 162:345–51. 9886405

[37] Mahanty S Mollis SN Ravichandran M Abrams JS Kumaraswami V Jayaraman K High levels of spontaneous and parasite antigen-driven interleukin-10 production are associated with antigen-specific hyporesponsiveness in human lymphatic filariasis. J Infect Dis (1996) 173:769–73. 10.1093/infdis/173.3.769 8627051

[38] Metenou S Dembele B Konate S Dolo H Coulibaly SY Coulibaly YI At homeostasis filarial infections have expanded adaptive T regulatory but not classical Th2 cells. J Immunol (2010) (184):5375–82. 10.4049/jimmunol.0904067 20357251 PMC3407820

[39] Carvalho LH Sano G Hafalla JC Morrot A Curotto de Lafaille MA Zavala F. IL-4-secreting CD4+ T cells are crucial to the development of CD8+ T-cell responses against malaria liver stages. Nat Med (2002) 8:166–70. 10.1038/nm0202-166 11821901

[40] Morrot A Hafalla JC Cockburn IA Carvalho LH Zavala F. IL-4 receptor expression on CD8+ T cells is required for the development of protective memory responses against liver stages of malaria parasites. J Exp Med (2005) 202:551–60. 10.1084/jem.20042463 16087712 PMC2212849

