# Supplementary figures and images for: Long-term Maintenance of CD4 T Cell Memory Responses to Malaria Antigens in Malian Children Coinfected with Schistosoma haematobium
Source: Front Immunol. 2018 Feb 1;8:1995. doi: 10.3389/fimmu.2017.01995 (PMC5799235; doi:10.3389/fimmu.2017.01995)

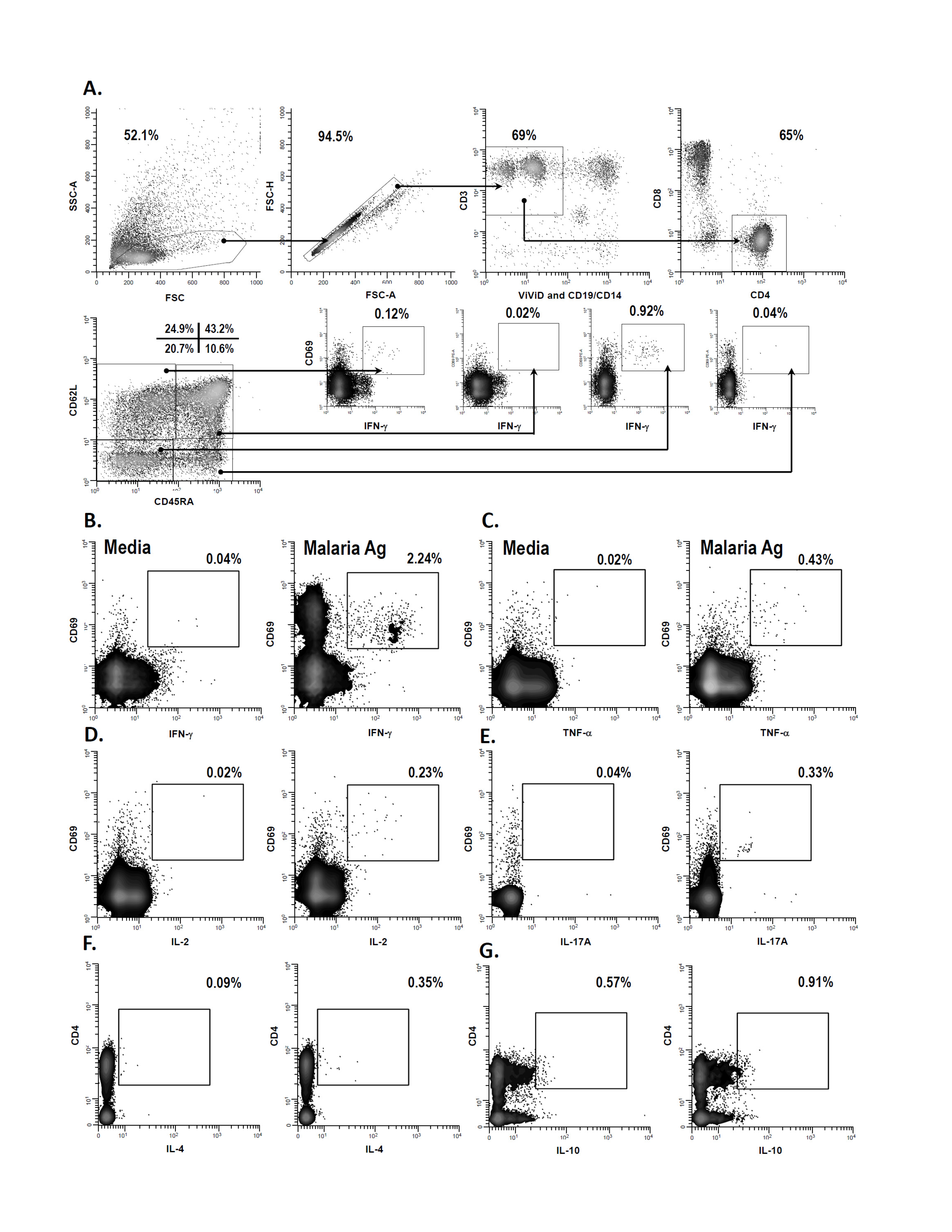

Supplement: Figure S1 — Gating strategy. Gating strategy for CD4+ memory response depicting (A) representative IFN-γ cytokine expression in response to malaria antigen stimulation from the central memory (CD62L+CD45RA−), naïve (CD62L+CD45RA+), effector memory (CD62L−CD45RA−), and EMRA (CD62L−CD45RA+) subpopulations. The media control and the malaria antigen-stimulated peripheral blood mononuclear cells (PBMC) from schistosoma-positive children and gated on CD19−CD14−CD8−CD4+ T cells after live/dead discrimination (B–G). Panels (B–E) depict CD69+ cytokine expression. Panels (F,G) are from an alternate panel and represent the total cytokine expressed from CD4+ T cells. [file image_1.tiff]
