# Supplementary material for: Long-term Maintenance of CD4 T Cell Memory Responses to Malaria Antigens in Malian Children Coinfected with Schistosoma haematobium
Source: Front Immunol. 2018 Feb 1;8:1995. doi: 10.3389/fimmu.2017.01995 (PMC5799235; doi:10.3389/fimmu.2017.01995)
Supplement: Supplementary file 4 [file table_1.docx]

**Table S1:** **Secondary Subset Demographics.** Demographic characteristics at enrollment and features of subsequent *P. falciparum* malaria infections of *Schistosoma haematobium*-positive (SP Mal) and age-matched *S. haematobium*-negative (SN Mal) Malian children contributing PBMC for secondary immunologic analysis.

| **Category** | **SP Mal**  **(n = 29) ^b^** | **SN Mal**  **(n = 29) ^b^** | **P value** |
| --- | --- | --- | --- |
| **Mean age (range)** | 8.7 (4-13) | 7.9 (4-13) | 0.26 |
| **Female (%)** | 15 (37.5) | 15 (51.7) | 0.09 |
| **Eggs (range) ^a^** | 28 (2-136) | 0 | n/a |
| **Malaria episodes (range)** | 1.55 (1-4) | 2.24 (1-5) | **0.019** |
| **Days to first malaria episode (range)** | 84.4 (11-174) | 21.5 (1-120) | **<0.0001** |
| **Parasitemia^c^ (range)** | 4,449 (750-263,000) | 4,356 (400--111,125) | 0.18 |

**^a^** Urinary egg excretion detected in 10 ml of filtered morning (10 am to 2 pm) urine.

**^b^** Excluding two children (one SP and one SN) with non-viable PBMC.

**^b^** Geometric mean parasite density per mm^3^
